# Role of novel histone modifications in cancer

**DOI:** 10.18632/oncotarget.23356

**Published:** 2017-12-17

**Authors:** Muthu K. Shanmugam, Frank Arfuso, Surendar Arumugam, Arunachalam Chinnathambi, Bian Jinsong, Sudha Warrier, Ling Zhi Wang, Alan Prem Kumar, Kwang Seok Ahn, Gautam Sethi, Manikandan Lakshmanan

**Affiliations:** ^1^ Department of Pharmacology, Yong Loo Lin School of Medicine, National University of Singapore, Singapore, Singapore; ^2^ Stem Cell and Cancer Biology Laboratory, School of Biomedical Sciences, Curtin Health Innovation Research Institute, Curtin University, Perth, WA, Australia; ^3^ Department of Botany and Microbiology, College of Science, King Saud University, Riyadh, Kingdom of Saudi Arabia; ^4^ Division of Cancer Stem Cells and Cardiovascular Regeneration, School of Regenerative Medicine, Manipal Academy of Higher Education (MAHE), Bangalore, India; ^5^ Cancer Science Institute of Singapore, National University of Singapore, Singapore, Singapore; ^6^ Curtin Medical School, Faculty of Health Sciences, Curtin University, Perth, WA, Australia; ^7^ National University Cancer Institute, National University Health System, Singapore, Singapore; ^8^ Department of Biological Sciences, University of North Texas, Denton, Texas, USA; ^9^ College of Korean Medicine, Kyung Hee University, Dongdaemun-gu, Seoul, Korea; ^10^ Institute of Molecular and Cell Biology, A*STAR, Biopolis Drive, Proteos, Singapore, Singapore; ^11^ Department of Pathology, National University Hospital Singapore, Singapore, Singapore

**Keywords:** cancer, histones, oncogenes, tumor suppressor genes, ubiquitination

## Abstract

Oncogenesis is a multistep process mediated by a variety of factors including epigenetic modifications. Global epigenetic post-translational modifications have been detected in almost all cancers types. Epigenetic changes appear briefly and do not involve permanent changes to the primary DNA sequence. These epigenetic modifications occur in key oncogenes, tumor suppressor genes, and transcription factors, leading to cancer initiation and progression. The most commonly observed epigenetic changes include DNA methylation, histone lysine methylation and demethylation, histone lysine acetylation and deacetylation. However, there are several other novel post-translational modifications that have been observed in recent times such as neddylation, sumoylation, glycosylation, phosphorylation, poly-ADP ribosylation, ubiquitination as well as transcriptional regulation and these have been briefly discussed in this article. We have also highlighted the diverse epigenetic changes that occur during the process of tumorigenesis and described the role of histone modifications that can occur on tumor suppressor genes as well as oncogenes, which regulate tumorigenesis and can thus form the basis of novel strategies for cancer therapy.

## INTRODUCTION

Post translational modifications (PTMs) on histone as well as non-histone proteins regulate gene expression profiles through chromatin structure alterations. These events often dictate key cellular events such as mitosis, meiosis, DNA damage response, gene expression, cell cycle, cell signaling pathways, energy, and metabolic pathways. PTMs such as phosphorylation, N-terminal acetylation, methylation, sumoylation, ubiquitination, propionylation, butyrylation, carbonylation, neddylation, proline isomerization, glycosylation, citrullination and poly ADP ribosylation regulate diverse protein functions [1, 2]. Epigenetic modifications in chromatin’s structure affect gene expression, such that the loosely packed form of DNA, which is called euchromatin, shows higher transcriptional activity; in contrast, the tightly packed form of DNA, heterochromatin, presents lower transcriptional activity [3]. Chromatin remodeling complexes can alter the nucleosome structure thereby modifying DNA accessibility [4]. Chromatin-modifying complexes can be divided into the adenosine triphosphate (ATP)-dependent complex and covalent histone modifications. Various ATP-dependent nucleosome-remodeling complexes have been reported in the literature, suggesting different mechanisms of action involved in histone sliding, ejection or the incorporation of histone variants [5]. The ATP dependent chromatin remodeling complex is divided into four subfamilies, 1) switch/sucrose non-fermentable (SWI/SNF), 2) chromo domain helicase DNA-binding (CHD), 3) imitation switch (ISWI), 4) INO80 [6]. In addition, several subtypes of chromatin remodelers have been identified that are involved in DNA damage, apoptosis and developmental pathways [7, 8]. A range of enzymes including histone lysine acetyltransferases (KATs), histone lysine deacetylases (KDACs), histone lysine methyltransferases (KMTs) and histone lysine demethylases (KDMs) regulate histone modifications [9-11]. Recent studies have shown that long noncoding RNAs are also involved in histone-modifying activities through histone H3 lysine 4 trimethylation [12]. Acetylation of several proteins is often found and regulated a plethora of cellular functions further emphasizing the importance of this PTM. Mass spectrometry analysis of cellular proteins revealed that acetylation occurred across different compartments of cells and was not confined to the nucleus of the cell [13]. PhosphoSitePlus(®) (PSP, http://www.phosphosite.org/), a knowledgebase dedicated to mammalian PTMs, reports that over 330,000 non-redundant PTMs, including phospho, acetyl, ubiquityl and methyl groups [14, 15].

Aberrant histone modifications are known to play a key role in the pathogenesis of several human diseases such as cancer, inflammatory and neurodegenerative diseases. Cancer-specific telomerase reverse transcriptase gene (*TERT*) promoter mutations (-146C>T and -124C>T) have been linked to reactivation of the epigenetically silenced *TERT*. *TERT* promoter mutations in human embryonic stem cells and their differentiation into fibroblasts and neural progenitor cells revealed that these mutations were capable of overcoming epigenetic silencing [16-20]. The reduction of H3K4me3 and H3K9ac levels at mutant *TERT* promoters was similarly observed in BRAF-mutant, *TERT* (−146C > T) or *TERT* (−124C > T) melanoma cell lines, UACC257, A375, WM793, and Malme-3M, following si-BRAF treatment [20]. These data collectively demonstrate the critical role of RAS-ERK signaling in regulating the active chromatin state of mutant *TERT* promoters in BRAF/NRAS-mutant human melanoma [20]. Various cytokines such as interleukin 1β (IL-1β), tumor necrosis factor α (TNF-α), lipopolysaccharides and other stimulants can promote histone acetylation. KATs activate inflammatory genes, whereas KDACs repress the inflammatory gene expression. Promoters of pro-inflammatory cytokines (such as IL-1, IL-2, IL-8, and IL-12) are rapidly acetylated and become transcriptionally active. KDACs regulate transcription of both pro-inflammatory and anti-inflammatory cytokines via their co-repressor complexes and transcription factors such as forkhead box P3 (FOXP3), signal transducer and activator of transcription (STATs), GATAs, zinc finger E-Box binding homeobox 1 (ZEB1), and nuclear factor - κB (NF-κB [21, 22]. Furthermore H3tre11 is a specific substrate for tumor specific pyruvate kinase M2 (PKM2) in EGF mediated transcription initiation and H3K9 acetylation, thus leading to tumor cell proliferation [23]. In addition to nuclear function, histones also act like an endogenous signal when they locate at the extra-nuclear space. As a response to stress, immune cells, cerebellar neurons, Schwann cells, and microglia present H2A, H2B, H3, H4, and H1 on their cell surface or cytoplasm. The levels of circulating histones as well as nucleosomes are increased in cancer, inflammation and infection, which suggest that histones could be potentially useful as biomarkers and therapeutic targets for these diseases [24-27].

## EPIGENETIC CHANGES IN TUMOR SUPPRESSOR GENE AND ONCOGENE

### Tumor suppressor gene *p53*

The tumor suppressor gene *p53* is the most widely studied gene that, under normal physiological conditions, regulates cell cycle, senescence, and differentiation, and induces apoptosis [28]. *p53* is often mutated, leading to uncontrolled cell proliferation in a variety of cancers [29]. One of the most frequently observed post-translational modifications is acetylation. Acetylation regulates both the loss of function and gain of function of *p53* such as enhancing DNA binding and preventing non-specific binding by the *p53* C terminal domain [30]. *p53* is acetylated by p300 at 5 lysine residues at the C-terminal regulatory region [31]. Ultraviolet light, ionizing radiation and activated *Ras* oncogene and other stress factors induce acetylation of *p53* [32, 33]. Interestingly, acetylation of *p53* blocks its ubiquitination. PCAF (P300/CBP-associated factor) acetylation of *p53* at K320 prevents ubiquitination by E4F1 [34]. Remarkably, both acetylation and prevention of ubiquitination activate *p53*. Ubiquitination of K370, K372, K373, K381, and K382 activates *p53* nuclear translocation and its subsequent proteosomal degradation [35]. Therefore, the acetylation event stabilizes *p53* and actively promotes its nuclear localization. Notably, p300 and PCAF acetylation sites are not mutated, except K120, which is mutated in cancers and is linked to cell cycle control and apoptosis mechanisms [28, 36-38]. TIP60, and MOF (males absent on the first)-two members of the MYST family- acetylate K120 and are located within the DNA binding domain of *p53* [36, 37]. K120 acetylation by TIP60 and MOF induces apoptosis with minimal to no effect on cell cycle genes. Acetylation of *p53* neutralizes the positive charge on the lysine residue thereby impairing its ability to form hydrogen bonds [35]. Acetylation also creates docking sites for transcriptional coactivators. For example, acetylation of K382 increases *p53* affinity to the CBP bromodomain [39, 40]. In contrast, deacetylation of both K373 and K382 enhances interaction with tandem bromodomains of TAF1, a TFIID subunit [40]. PC4 is a transcription factor that is acetylated by p300. It has been shown that PC4 interaction with *p53* leads to acetylation of both the proteins and enhances the *p53* DNA binding ability and expresses *p53* regulated gene products, especially during DNA damage [41, 42].

### Oncogene *cMYC*

One of the most commonly activated oncogenes in cancer cells is *Myc*, which regulates the complex inflammatory response [33, 43]. In B-cells, activation of *Myc* rapidly induces the synthesis and release of IL1-β [36]. The pleiotropic effects of oncogenes include creating a pro-tumor microenvironment and a persistent, constitutive activation of pro-inflammatory transcription factors [44-46]. The involvement of human c-Myc in cancer progression is shown in multiple cancer types, including Burkitt’s lymphoma, multiple myeloma and T cell leukemia [47]. cMyc is deregulated and abnormally overexpressed in a variety of cancers [43]. Wasylishen AR et al., 2014 showed that *Myc* signaling is regulated by six lysine residues located at the highly conserved *Myc* homology box IV. These lysine residues negatively regulate *Myc* induced transformation and tumorigenesis, and thus serve as potential targets for cancer therapy [48]. This lysine rich region has previously been associated with *Myc* acetylation [49, 50]. *Myc* also serves as a substrate for KATs which modify different lysines. The following lysines, 143, 148, 157, 275, 317, 323, 371, and 417, are acetylated either in response to co-expression of mGCN5 or p300 or by *in vitro* acetylation with p300 [51]. Other studies indicated that knocking down JMJD1A, a histone demethylase inhibited the proliferation and survival of prostate cancer cells. . This study unveils the prime role of JMJD1A in upregulating c-Myc expression by AR (androgen receptor) dependent mechanism *via* H3K9 demethylation activity and controlling post transcriptional activity of c-Myc [52]

## EPIGENETIC ORIGIN OF SPORADIC CANCERS

Genetic and environmental inter-relationships play a vital role in the development of sporadic forms of cancers and are often associated with genetic alterations in these tumors [53]. For instance, breast cancer and its subtypes can be differentiated based on their genomic and epigenetic aberrations. DNA methylation is often observed in both normal as well as in cancerous tissues. Several hundred genes are either hypermethylated or hypomethylated in breast cancer [54]. Epigenetic silencing or deletion of *BRCA1* is commonly observed in sporadic breast cancer [55]. Familial CRC occurs in 15% of cases, hereditary in 5% of cases, and sporadic in almost 75-80% of cases. Several somatic mutations have been in identified in the tyrosine phosphatome, and PTPRG, PTPRT, PTPN3, PTPN13, and PTPN14 were all associated with loss of function [53]. Genetic and environmental factors play key roles in the susceptibility of individuals to sporadic forms of CRC resulting in loss of protein function.

## HISTONE PHOSPHORYLATION IN CANCER

**Figure 1:**
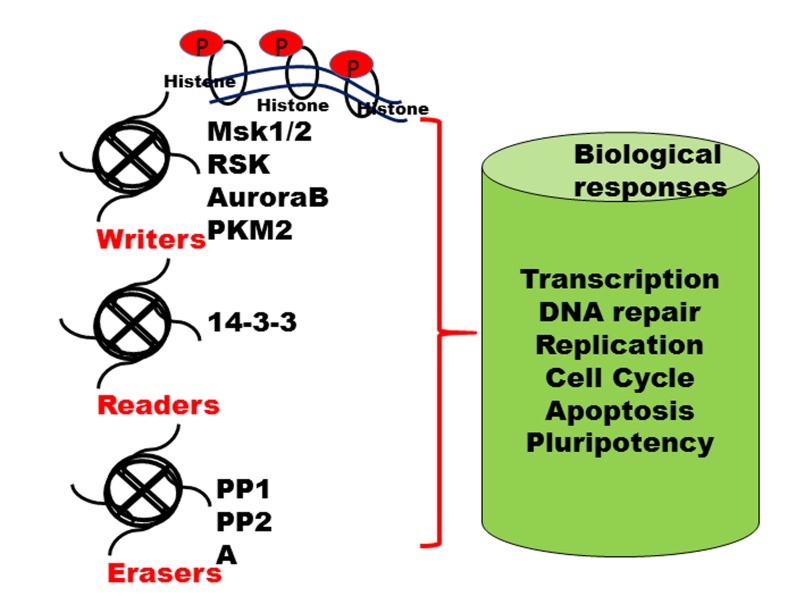
Writers, readers and erasers of histone marks Reversible PTMs addition, identification and removal of these modifications on histone tails regulate various biological processes, including transcription, DNA replication and DNA repair and are highly dynamic in nature. The "writers" such as histone protein kinases add specific PTMs on specific amino acid residues on core histone tails. These marks are identified by specific protein domains called "readers" such as 14-3-3 proteins. The PTM marks are removed by active enzymes known as "erasers" such as phosphatases; Abbreviations: RSK, ribosomal protein S6 kinase A3; AuroraB, serine/threonine protein kinase Aurora B; PKM2, pyruvate kinase M2; Msk1/2, Mitogen- and stress-activated protein kinase 1 and 2, PP, protein phosphatases.

Histone phosphorylation is one of the PTM events that take place during DNA damage, activation of transcription and chromatin remodeling/compaction during cell division and apoptosis, and other nuclear processes. Histone phosphorylation is regulated by various kinases and phosphatases [56]. These kinases and phosphatases can potentially act on PTM sites on any of the four nucleosomal histone tails. Traditionally, phosphorylation occurs on tyrosine, serine, and threonine residues [57, 58]. Several phosphorylation sites have been identified in histone tails that regulate nuclear processes. During DNA damage, phosphorylation of core histone H2A(X) is a key event; serine 139 gets phosphorylated on the H2A(X) variant histone, commonly referred to as γH2A(X) [59-63]. This phosphorylation occurs during cell cycle and DNA damage response, and is mediated by protein kinases yTel1 and yMec1 (ATM and ATR in mammals) in yeast [64, 65]. Importantly, histone phosphorylation is associated with transcriptional regulation and gene expression, especially genes that regulate cell cycle and proliferation [60]. Phosphorylation of serine (Ser) 10 and 28 on H3, and serine 32 on H2B have been associated with activation of epidermal growth factor (EGF) mediated gene transcription. This phenomenon has also been observed upon exposure to ultraviolet B radiation, whereH3ser10p and H2Bser32p upregulated the expression of proto-oncogenes such as *c-myc, c-fos, c-jun* [66-68]. Furthermore, H3ser28p regulated the activation of c-fos and α-globin [69]. Phosphorylation of H3 at ser10, 28 and threonine (Thr) 6 and 11 has been shown to occur upon androgen stimulation and DNA damage. [56, 70-73]. Taken together, a complex crosstalk between phosphorylation events leads to gene expression, and cell proliferation. H2Bser32p occurs ubiquitously in normal human cells; however, it is also extensively phosphorylated by RSK2 kinase (an AGC kinase of the RSK family of kinases) in skin cancer cells [67, 74]. Several protein kinases have been associated with phosphorylation of histones such as RSK2, MSK1/2, and mixed lineage kinase-like mitogen-activated protein triple kinase alpha (MLTK)-α [56]. In addition, androgen stimulation protein kinase C-beta (PKCβ) and protein kinase C related kinase 1 (PRK1) phosphorylate H3tre6 and 11 respectively in prostate cancer cells [70, 71]. Histone tyrosine (tyr) phosphorylation is also associated with transcription initiation. Janus kinase 2 (JAK2) has also been shown to phosphorylate H3tyr41, thereby disrupting chromatin binding by heterochromatin protein 1α (HP1α). HP1α has been shown to directly interact with H3 through its chromo-shadow domain. Thus, loss of HP1α from chromatin leads to constitutive activation of the JAK2 signaling pathway including oncogene *imo2*, leading to oncogenesis [75]. Several studies indicated that Aurora B was overexpressed in a variety of human cancers particularly in colorectal and breast cancer, and critical for H3 phosphorylation and for accurate chromosome segregation [76-79]. Remarkably, H2AX phosphorylation increases several fold upon DNA fragmentation and apoptosis [80, 81]. Mst1 kinase has been shown to phosphorylate H2AX. Overexpression of Mst1-induced apoptosis of HELA cells is associated with H2AXser139p [82] (Figure 1) (Table 1).

**Table 1:** Transcriptional and cellular role of novel histone modifications in cancers

Post-translational Modifications	Histone residues modified	Role in cell activity and transcription	Enzymes	References
Phosphorylation(serine, threonine, tyrosine)	H3 (Ser10, 28; Thr 3, 6, 11,45 and Tyr41) H2B (Ser32) Histone variant H2A(X)	Activation DNA damage response	ATM, ATR, RSK2, PKM2, Aurora B kinase.	[23, 56, 64, 65, 67, 68, 72]
Lysine ubiquitination	H2A (K119) H2B (K120)	Transcription regulation	E2A ubiquitinase	[104, 109]
p53 acetylation and ubiquitination	K370; K372; K373; K381; K382; K120	Proteasomal degradation; apoptosis	p300/PCAF; TIP60; MOZ;	[35, 39, 40]

## HISTONE POLY-ADP RIBOSYLATION IN CANCER

Poly-ADP ribosylation is involved in the development and progression of cancer thorough diverse pathways. Involvement of mono and/or poly-ADP-ribosylation has been shown to be involved in carcinogenesis and has been widely implicated in many cancers including hepatocellular carcinomas, lymphomas, endometrial, oral and colon cancers [83-88]. Poly-ADP-ribose-polymerase (PARP) and poly-ADP-ribose-glycohydrolase (PARG) function as DNA repair enzymes that play prominent roles in the base-excision repair and double strand break repair pathways [89]. The primary prerequisite in DNA repair of heterochromatic DNA is chromatin relaxation to allow DNA repair enzymes access to DNA lesion sites. Several studies have demonstrated that PARP-1 mediated poly-ribosylation is an essential component of different repair pathways such as base excision repair (BER) [90], nucleotide excision repair [91], homologous recombination [92], and non-homologous end joining [93]. PARP-1 also has a role in epigenetic regulations that promote oncogenesis [85]. Cohen-Armon *et al.*, 2007 showed that phosphorylated ERK2 activates PARP-1 and Elk1, thereby promoting CBP/p300 dependent acetylation and transcription of Elk1 target genes [94]. In another study, CDK1 dependent phosphorylation of PARP-1 displaced histone H1 from chromatin, thus accelerating progestin-mediated breast cancer cell proliferation [95]. PARP-1 mediated PARylation of the lysine demethylase, KDM5B, inhibited binding to its target proteins and maintained the H3K4me3 methylation status in MCF7 breast cancer cells [96]. The transient ectopic overexpression of CCCTC-binding factor (CTCF) induces the PARP-1/CTCF (CCCTC binding factor) complex that suppresses DNA methyltransferase 1 (DNMT1)-mediated DNA methylation-dependent silencing and chromatin compaction [97, 98]. Furthermore, PARP-1 deficiency induces differentiation of embryonic stem cells to syncytiotrophoblastic giant cells and produces tumors phenotypically similar to teratocarcinoma [99, 100].

## HISTONE UBIQUITINATION AND TRANSCRIPTIONAL REGULATION

Epigenetic modifications are regulated by PTMs, including ubiquitination, and can directly modulate either histones or non-histone proteins such as transcription factors or their co-factors. The balance between ubiquitination and deubiquitination is important for cellular homeostasis, and any imbalance in this equation often leads to tumorigenesis. Ubiquitin is a 76-amino acid polypeptide containing seven lysine (K) residues with poly-ubiquitin chains linked through the K6, K11, K27, K29, K33, K48, and K63 residues Ubiquitin contains. The process of ubiquitination (attachment of ubiquitin to a histone protein) is an ATP dependent process activated by an E1 enzyme, followed by an E2 class enzyme allowing E3 ubiquitin ligase to ubiquitinate target proteins directly or indirectly [101-103]. The target protein can either be mono or poly-ubiquinated and the consequence depends on the type of chains formed [104]. Distinct mono and poly-ubiquitination are known to regulate target protein activity [105]. For instance, K6 and K48 poly-ubiquitin chains regulate proteosomal degradation, while K11 targets proteins degradation *via* endoplasmic reticulum mediated d pathways. K11 is also involved in regulation of cell cycle progression while K29 is involved in protein degradation by lysomes [106, 107]. Histone ubiquitination is implicated in several conserved cellular processes such as DNA repair, transcriptional regulation, and genome stability. Unlike other proteins, histone poly-ubiquitination does not mark histones for degradation by 26s proteasomes, but rather is associated with transcriptional regulation [108]. One major PTM is histone H2A ubiquitination, which plays a role in transcriptional silencing by polycomb proteins and genome maintenance. H2A is the most abundant histone fraction to be mono-ubiquitinated at K119 compared to H2B at K120. These ubiquitination marks can be deubiquitinated by MYSM1 (2A-DUB) [109]. All the four core histones H2A, H2B, H3, and H4 become mono-ubiquitinated upon DNA damage and are known ubiquitin substrates *in vivo* [106, 108]. H2B is usually monoubiquitinated while H2A can be mono or poly-ubiquitinated. Ubiquitination of the C-terminal tail of H2B promotes transcription elongation, disrupts chromatin structure and nucleosomes, and significantly increases transcript length [110, 111]. H2B monoubiquitination (H2Bub1) is a key PTM that plays an important role in both transcriptional activation and tumor suppression [112, 113]. Aberrant H2Bub1 ubiquitination and deubiquitination is the primary driver of tumorigenesis and directly impact upon chromatin structure beyond the single nucleosome level [114, 115]. H2B ubiquitination is regulated by ubiquitin-conjugating enzyme E2A (UBE2A or RAD6A), and the RNF20/RNF40 ubiquitin ligase complex. Aberrant expression of these enzymes often leads to the development and progression of several tumor types such as seen with hypermethylation of RNF20 promoters in breast tumor samples. Maintenance of H2B monoubiquitination is dependent on tumor suppressor cell division cycle 73 (CDC73). Mutation of CDC73 results in corresponding loss of H2B monoubiquitination in both *in vitro* and *in vivo.* Mutated or abnormally regulated CDC73 has been reported in parathyroid, renal, breast, gastric, and colorectal tumors, as well as in the germline of patients with the familial disorder-hyperparathyroidism jaw tumor syndrome [116]. Thus, altered expression levels of RNF20 contribute to the deferred H2Bub1 levels, leading to cancer development and progression. H2Bub1’s role in tumor suppression was correlated with decreased abundance in malignant breast cancer samples compared to normal and benign samples [117]. Aberrant expression and over-activation of deubiquitination enzymes also affect the global abundance of H2Bub1 [115, 118, 119]. Specific mutations such as non-synonymous single nucleotide polymorphisms, amplifications, and homozygous deletions in deubiquitinase USP22 have been reported in a variety of tumor types [108]. In a study by Zhang *et al.*, USP22 was upregulated in breast cancer samples obtained from patients with aggressive phenotypes compared to benign tumors, and correlated with a decrease in H2Bub1 levels [120]. Furthermore, USP22 overexpression correlated with lymph node metastasis and breast cancer reoccurrence, and was an indicator of poor prognosis [120, 121].

## HISTONE SUMOYLATION IN CANCER

Similar to other reversible post-translational modifications, sumoylation of histones is characterized by covalent addition of small ubiquitin-like modifier (SUMO) to a target protein. It is one of the most essential post translational modifications and plays a key role in various physiological and pathological processes. SUMO exists in four isoforms named SUMO-1, -2, -3, -4 [122] which are 12 kDa in size, and share a structural similarity with ubiquitin [123-126]. Sumoylation of protein does not lead to proteosomal degradation; in contrast it is required for protein stability, transcription regulation of glucocorticoid receptor, Myb, CAAT/enhancer binding protein (C/EBP) and SP3. Sumoylation is a negative regulator and attenuates transcriptional activity [127, 128]. Desumoylation is mediated by specific cysteine-protease Sentrin-specific protease (SENP) which removes the sumoylation marks on proteins [129]. Several transcription factors like activating protein 2 (AP-2), androgen receptor (AR), c-Jun, c/EBP, c-Myb, CREB, GATA-2, heat shock factor (HSF1), interferon regulatory factor (IRF-1), p53, Sp3, STAT1, have been demonstrated to be SUMOylated in cancer [130-132]. Emerging evidence indicated that sumoylation is also known to regulate the enzymatic activity of histone modifying enzymes. Several histone modifying enzymes such as HDAC1, HDAC2, HDAC4, SIRT1, EZH2 and KDM5B are known to be modified by SUMO thereby linking SUMOylation to epigenetic regulation [133-137].

## HISTONE GLYCOSYLATION IN CANCER

Several other PTMs of histone have been identified which are associated with the regulation of histone function and chromatin remodeling. Core histones H2A, H2B, H3 and H4 were found to be glycosylated to O-linked N-acetylglucosamine (O-GluNAc) modification on serine and threonine residues such as H2A at T101, H2B at S36, H3S10 and T32 and H4 at S47 [138-142]. This modification is mediated by two enzymes, O-GlcNAc transferase (OGT) and O-GlcNAc hydrolase (OGA). Both histones and cytosine demethylases are modified by O-GlcNAc in a variety of cancer cells [139, 141-143]. Over expression of OGT crosstalk with other histone PTM such as H3K9 acetylation, H3S10 phosphorylation, and H3R17/K27 methylation suggesting that O-GlcNAc signaling might regulate chromatin dynamics by affecting other histone marks [140, 141, 144]. Other studies have demonstrated the role of histone glycosylation in normal and cancer cells. This study indicated the early reduction of OGlcNAcylation level at H3 on serine 10 and 28 in S phase whereas elevated levels was observed in G2/M phase. However, the function and mechanism of histone O-GlcNAcylation and other histone modifications such as propionylation, butyrylation, carbonylation, citrullination and proline isomerization still remain to be elucidated.

## HISTONE NEDDYLATION IN CANCER

Similar to the ubiquitin pathway, ubiquitin-like protein NEDD8 has been identified as targeting proteins for degradation via the neddylation pathway [145]. NEDD8 is 80% homologus to ubiquitin and modulates ubiquitin induced protein degradation. Unlike ubiquitin, NEDD8 conjugated proteins are more abundant in the nucleus than in the cytoplasm [146, 147]. The most commonly investigated protein substrates of neddylation are Cullin-RING ligases. These are conjugated to NEDD8 via its C-terminal Gly-76 by NEDD8 [147-149] and deneddylated by COP9 signalosome (CSN) [150] Interestingly, it was observed that the cellular stress condition leads to drastic increase in neddylation. However the reason for this phenomenon is still unclear. Several key molecules involved in tumorigenesis are known to be regulated by neddylation and ubiquitination. In a majority of sporadic renal cancers, mutation in VHL protein is often observed and this is a substrate for NEDD8 [150-152]. In high grade neuroendocrine tumors, high levels of neddylated culin-1 is associated with tumor progression [153]. Squamous Cell Carcinoma-related Oncogene SCCRO (also known as DCUN1D1) is a part of E3 ligase complex (Cul-1-ROC1-SCCRO) is associated with the development of primary of squamous cell carcinoma of the lung with malignant phenotypes [154, 155]. Several other substrates of neddylation have also been observed in cancers. The RNA binding protein Hu antigen R (HuR) regulates cell dedifferentiation, proliferation and survival is neddylated in hepatocellular and colorectal carcinoma [156]. Other studies indicated that nedyylation of p53 by FBXO11, a member of the F-box protein family suppressed the function of p53 in lung cancer models [157]. In gastrointestinal cancers, neddylation of NF-κB upstream kinase IKKγ leads to activation of NF-κB [158]. In several cancers Mdm2, a E3 ubiquitin protein ligase that is involved in the ubiquitination and degradation of tumor suppressor protein p53 is also neddylated and promotes conjugation of NEDD8 to p53 [159].

## CONCLUSION AND PERSPECTIVES

Modifications in the tail regions and globular domains of histones can produce multifaceted consequences to cellular process such as transcription and replication, or even lead to malignant transformations. Several important histone modifiers have been identified and have been extensively discussed in this review. Histone acetylation is a mark associated with an active chromatin state. PTMs of histones such as histone acetyltransferases and deacetylases have been identified in the initiation, progression, and metastasis of cancers and some of these events are indicators of poor prognosis. Several novel histone post-translational modifications have been a subject of intense study for their role in tumor suppression, and also some exhibiting oncogenic functions. In addition, phosphorylation of histone by histone kinases, poly-ADP ribosylation, and histone ubiquitination have been implicated in the activation of multi-step and diverse pathways in transcription regulation and in the development of cancer. Therefore, histone modifications can be drivers of transcriptional change with relevant functions, rather than being mere by-products of PTMs or markers of the chromatin state. More work is needed to untangle the role of PTMs occurring at the same residue with contrasting functions. Several new epigenetic targets have also been identified with vast potential in epigenetic-specific therapy. Recent advances in epigenetics research have identified novel modifications in the core histones that have not yet been fully characterized. Overall, additional work is required to decipher the role of novel histone acylation, butyrylation, propionylation, and crotonylation, which will provide insight and extend our knowledge regarding chromatin compaction, transcription regulation, and the development of a variety of diseases including hematological malignancies, organ specific cancers, tumor metastasis, DNA repair, cellular reprogramming, differentiation, and pluripotency.
