# Correction: Role of novel histone modifications in cancer

**DOI:** 10.18632/oncotarget.25152

**Published:** 2018-04-10

**Authors:** Muthu K. Shanmugam, Frank Arfuso, Surendar Arumugam, Arunachalam Chinnathambi, Jinsong Bian, Sudha Warrier, Ling Zhi Wang, Alan Prem Kumar, Kwang Seok Ahn, Gautam Sethi, Manikandan Lakshmanan

**Affiliations:** ^1^ Department of Pharmacology, Yong Loo Lin School of Medicine, National University of Singapore, Singapore, Singapore; ^2^ Stem Cell and Cancer Biology Laboratory, School of Biomedical Sciences, Curtin Health Innovation Research Institute, Curtin University, Perth, WA, Australia; ^3^ Department of Botany and Microbiology, College of Science, King Saud University, Riyadh, Kingdom of Saudi Arabia; ^4^ Division of Cancer Stem Cells and Cardiovascular Regeneration, School of Regenerative Medicine, Manipal Academy of Higher Education (MAHE), Bangalore, India; ^5^ Cancer Science Institute of Singapore, National University of Singapore, Singapore, Singapore; ^6^ Curtin Medical School, Faculty of Health Sciences, Curtin University, Perth, WA, Australia; ^7^ National University Cancer Institute, National University Health System, Singapore, Singapore; ^8^ Department of Biological Sciences, University of North Texas, Denton, Texas, USA; ^9^ College of Korean Medicine, Kyung Hee University, Dongdaemun-gu, Seoul, Korea; ^10^ Institute of Molecular and Cell Biology, A*STAR, Biopolis Drive, Proteos, Singapore, Singapore; ^11^ Department of Pathology, National University Hospital Singapore, Singapore, Singapore

**This article has been corrected:** The correct author name is given below:

**Jinsong Bian**

Original article: Oncotarget. 2018; 9:11414-11426. https://doi.org/10.18632/oncotarget.23356

